# Higher Level Phylogeny and the First Divergence Time Estimation of Heteroptera (Insecta: Hemiptera) Based on Multiple Genes

**DOI:** 10.1371/journal.pone.0032152

**Published:** 2012-02-27

**Authors:** Min Li, Ying Tian, Ying Zhao, Wenjun Bu

**Affiliations:** 1 Institute of Entomology, College of Life Sciences, Nankai University, Tianjin, China; 2 Patent Examination Cooperation Center, State Interllectual Property Office of the Peoples' Republic of China, Beijing, China; Brigham Young University, United States of America

## Abstract

Heteroptera, or true bugs, are the largest, morphologically diverse and economically important group of insects with incomplete metamorphosis. However, the phylogenetic relationships within Heteroptera are still in dispute and most of the previous studies were based on morphological characters or with single gene (partial or whole 18S rDNA). Besides, so far, divergence time estimates for Heteroptera totally rely on the fossil record, while no studies have been performed on molecular divergence rates. Here, for the first time, we used maximum parsimony (MP), maximum likelihood (ML) and Bayesian inference (BI) with multiple genes (18S rDNA, 28S rDNA, 16S rDNA and COI) to estimate phylogenetic relationships among the infraorders, and meanwhile, the Penalized Likelihood (r8s) and Bayesian (BEAST) molecular dating methods were employed to estimate divergence time of higher taxa of this suborder. Major results of the present study included: Nepomorpha was placed as the most basal clade in all six trees (MP trees, ML trees and Bayesian trees of nuclear gene data and four-gene combined data, respectively) with full support values. The sister-group relationship of Cimicomorpha and Pentatomomorpha was also strongly supported. Nepomorpha originated in early Triassic and the other six infraorders originated in a very short period of time in middle Triassic. Cimicomorpha and Pentatomomorpha underwent a radiation at family level in Cretaceous, paralleling the proliferation of the flowering plants. Our results indicated that the higher-group radiations within hemimetabolous Heteroptera were simultaneously with those of holometabolous Coleoptera and Diptera which took place in the Triassic. While the aquatic habitat was colonized by Nepomorpha already in the Triassic, the Gerromorpha independently adapted to the semi-aquatic habitat in the Early Jurassic.

## Introduction

The hemipteran suborder Heteroptera are part of the most diversified group of non-endopterygote and nonholometabolous insects, including more than 40,000 described species [1].

**Figure 1:**
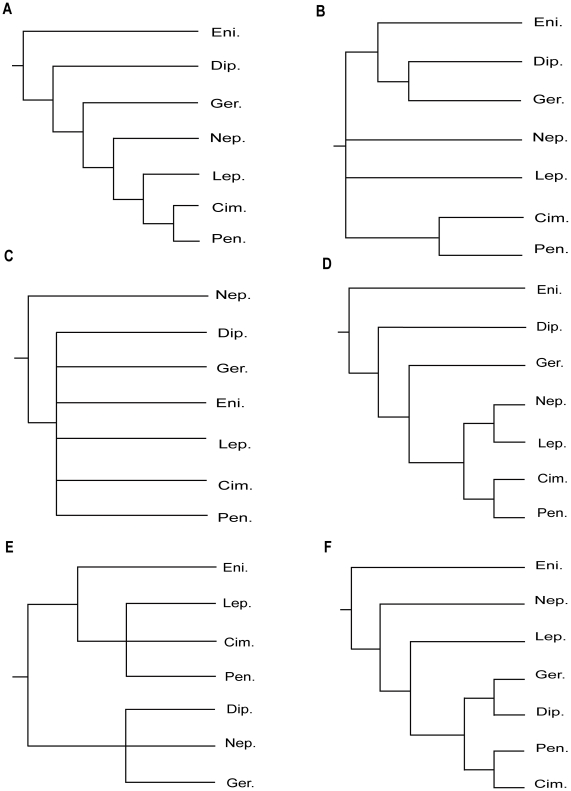
Proposed phylogenetic hypotheses within Heteroptera. (A) after Schuh (1979). (B) after Zrzavý (1992). (C) after Mahner (1993) & Shcherbakov and Popov (2002). (D) after Wheeler et al. (1993). (E) after Yang (2002). (F) after Xie et al. (2008). Cim.: Cimicomorpha; Dip.: Dipsocoromorpha; Eni.: Enicocephalomorpha; Ger.: Gerromorpha; Lep.: Leptopodomorpha; Nep.: Nepomorpha; Pen.: Pentatomomorpha.

Classification of the Heteroptera, or true bugs, has reached its present state through a long evolutionary process beginning, insofar as modern systematics is concerned, with the work of Linnaeus. In the system of Linnaeus, the true bugs were placed in the Hemiptera, the first recognized higher group in Insecta, also to include Thysanoptera (thrips), and the other hemipteran suborders Sternorrhyncha (aphids, coccoids) and Auchenorrhyncha (cicadas). All true bugs at that time were divided by Linnaeus into three genera: *Notonecta*, *Nepa*, and *Cimex*. These are all familiar modern-day generic names, but the concepts attached to them have become more restricted over time, particularly for *Cimex*. Latreille (1810) [2] formally named the subgroups Heteroptera and Homoptera in Hemiptera, and later [3] divided the Heteroptera into Hydrocorisae and Geocorisae based on the structure of the antennae. Geocorisae of Latreille was subsequently divided by Dufour (1833) [4] with recognizing of the Amphibicorisae (modern-day Gerromorpha) from it. Fieber (1861) [5] introduced the terms Gymnocerata and Cryptocerata for the Geocorisae and Hydrocorisae, respectively. The classic subdivisions of Latreille and Dufour had a profound impact on the Heteroptra and were used well into the twentieth century. Leston et al. (1954) [6] introduced the terms Cimicomorpha and Pentatomomorpha in the first formal attempt to recognize natural groups within the Geocorisae. Their work spurred the attempt to document the monophyly of higher groups within the Heteroptera, with the eventual recognition of seven such groups-termed infraorders all with typified names, as outlined by Štys and Kerzhner (1975) [7]. The seven infraorders are: Enicocephalomorpha, Dipsocoromorpha, Gerromorpha, Nepomorpha, Leptopodomorpha, Cimicomorpha and Pentatomomorpha. Explicit cladistic hypotheses based on molecular data (or molecular and morphological data) for relationships of Gerromorpha was Damgaard (2008) [8]; for Nepomorpha were Hebsgaard et al. (2004) [9] and Hua et al. (2009) [10]; for Cimicomorpha were Tian et al. (2008) [11]and Schuh et al. (2009) [12]; for Pentatomomorpha were Li et al. (2005) [13], Hua et al. (2008) [14] and Tian et al. (2011) [15], however, those for relationships among the seven infraorders were relatively rare. Schuh (1979) [16] firstly documented higher-level phylogenetic scheme of the Heteroptera in a cladistic context, with 11 characters of the first instar larvae and adult derived mostly from Cobben (1978) [17] and considered Enicocephalomorpha as the sister group of the remaining Heteroptera, with Leptopodomorpha+Nepomorpha as the sister group of Cimicomorpha+Pentatomomorpha, as shown in Figure 1A.

Then, several other studies on phylogeny of Heterotera based on morphological data have been undertaken. Zrzavý (1992) [18], based on the characters of antennal exoskeleton, indicated that Enicocephalomorpha and the sister group of Dipsocoromorpha+Gerromorpha together formed a basal heteropteran clade, however, the relationships among Nepomorpha, Leptopodomorpha, and Cimicomorpha+Pentatomomorpha were still unresolved (Figure 1B). Mahner (1993) [19] proposed a hypothesis that Cryptocerata (Nepomorpha) was placed as the sister taxon to the remaining Heteroptera based on morphological characters (see in Discussion) (Figure 1C). The results of Mahner (1993) [19] supported the treatment of Latreille (1825) [3] and Fieber (1861) [5] which divided the Heteroptera into two groups, Cryptocerata and Gymnocerata. Shcherbakov and Popov (2002) [20], based on fossil and Recent taxa and morphological evidence, presented an incertitude strictly cladistic scheme of relationships for the infraorders of Heteroptera, which was concordant with Mahner's hypotheses (Figure 1C). However, these two proposed hypotheses were not supported by the further studies based on morphological data and 18S rDNA sequences only [21]–[23].

Wheeler et al. (1993) [21] attempted firstly to combine molecular and morphological data to analyze the phylogenetic relationship for higher groups of Heteroptera. They used about 669 bp of 18S nuclear rDNA sequences and 31 morphological data for 29 hemipteran taxa, representing all infraorders and six outgroup taxa including the Psocoptera, Sternorrhyncha, Auchenorrhyncha, and Coleorrhyncha. The scheme, shown in Figure 1D, indicated substantial congruence between the molecular data and most of the morphological data used by Schuh (1979) [16]. However, the placements of Nepomorpha and Leptopodomorpha differed from each other (Figure 1A D). Yang (2002) [22] examined male genitalia of 300 species of 40 families and obtained the cladogram Enicocephalomorpha+(Leptopodomorpha+Cimicomorpha+Pentatomomorpha)+(Dipsocoromorpha+(Nepomorpha+Gerromorpha)), in which Enicophalomorpha was regarded as the sister group to the remaining Heteroptera (Figure 1E).

Recently, a novel hypothesis, based on whole sequences of 18S rDNA whose alignment were modified by the secondary structure of rRNA, with 26 representatives of Heteroptera and 11 outgroups, was proposed by Xie et al. (2008) [23]. All infraorders were monophyletic in their analyses, and infraordinal relationships were recovered as (Enicocephalomorpha+(Nepomorpha+(Leptopodomorpha ((Gerromorpha+Dipsocoromorpha)+(Cimicomorpha+Pentatomomorpha))))) (Figure 1F). Forero (2008) [24] performed a brief review on the main phylogenetic hypotheses for Hemiptera and its subgroups. Besides, Damgaard (2008) [8], using 64 morphological characters and ∼2.5 kb of DNA sequence data from the mitochondrial genes encoding COI+II and 16S rRNA and the nuclear gene encoding 28S rRNA, gave the relationship as (Enicocephalomorpha+(Dipsocoromorpha+(Gerromorpha+Nepomorpha))). Cassis & Schuh (2010) [25] presented a cladistic analysis of a comprehensive morphological data (including fossil and Recent taxa) for the Panheteroptera and the monophyly of (Leptopodomorpha+(Cimicomorpha+Pentatomomorpha)) was supported. Weirauch & Schuh (2011) [1] reviewed the systematics of Heteroptera comprehensively after the excellent overviews of the biology and taxonomy of this suborder done by Schuh & Slater (1995) [26].

Until now, the monophyly of Heteroptera and of each heteropteran infraorder, have been generally accepted [1], [16], [18]–[20], [22], [23]. A stable sister-group relationship between Cimicomorpha and Pentatomomopha has been supported by all the comprehensive studies [12], [16], [18], [21], [23], [25]. However, there are still certain things at variance concerning phylogenetic relationships within Heteroptera: 1) whether Enicophalomorpha was the sister taxa to the remaining Heteroptera, or the Nepomorpha (Cryptocerata) as proposed by Mahner (1993) [19]; 2) the positions of Dipsocoromorpha, Gerromorpha and Leptopodomorpha are not determined.

A unified understanding of 400 million years of insect evolution requires insight into their origin [27]. Divergence times and radiations of higher groups of Coleoptera [28] and Diptera [29] have been well estimated based on molecular data. Although the records of comparatively rich fossils demonstrated that the groups of Heteroptera at family level underwent a wide radiation in the Jurassic; Cimicomorpha and Pentatomomorpha underwent a major expansion in the Cretaceous (Gillott, 2005) [30], however, the divergence time of higher taxa of Heteroptera from molecular data remains very poorly resolved.

This work is the first attempt to apply multiple genes of nucleus and mitochondria to reconstruct the evolutionary relationships of higher groups within Heteroptera. The nuclear 18S ribosomal gene (18S rDNA), D3 region of 28S ribosomal gene (28S rDNA) and a portion of mitochondrial 16S ribosomal gene (16S rDNA) and Cytochrome Oxidase Subunit I (COI) were chosen as molecular markers. Also, according to relaxed clock models, the ages of the most recent common ancestors of higher groups of Heteroptera were estimated.

## Results

### Data characteristics

Nucleotide sequence editing and alignment resulted in a 18S rDNA data partition of 1982 characters, a 28S rDNA data partition of 607 characters, a 16S rDNA data partition of 530 characters, a COI data partition of 1067 characters; a nuclear data partition (18S rDNA+28S rDNA) of 2589 characters; a mitochondria data partition (16S rDNA+COI) of 1597 characters; and a total combined data set of 4186 characters. The detailed information for the sequences is in Table 1.

**Table 1:** Data statistics for individual and combined analyses.

	18S	28S	16S	COI	18S+28S	16S+COI	Combined
No. Char. (bp)	1982	607	530	1067	2589	1597	4186
Var. sites/Var.%	1185/59.8	341/56.2	404/76.2	629/61.2	1526/58.9	1033/64.7	2559/61.1
PIC./PIC. %	843/42.5	267/44.0	347/65.5	557/52.2	1110/42.9	905/56.7	2015/48.1
A%	25.2	24.7	31.0	32.0	25.1	31.7	27.7
C%	23.1	24.3	9.5	17.3	23.4	14.6	19.8
G%	27.1	30.0	17.3	16.4	27.8	16.7	23.3
T%	24.6	21.0	42.2	34.3	23.7	37.0	29.1
Ts∶Tv	1.0	1.1	0.5	0.7	1.0	0.7	0.8
Retention index(RI)	0.6148	0.6143	0.2710	0.2673	0.6163	0.1668	0.4427
Consistency index(CI)	0.3074	0.4706	0.2448	0.2226	0.3119	0.3167	0.2352

Note: No. Char. = total aligned number of characters; Var. sites = number of variable sites; Var. % = Var. sites/No. Char.;

PIC. = phylogenetically informative characters; PIC. % = PIC./No. Char.; Ts∶Tv = Transition/Transversion; RI = retention index;

CI = consistency index.

### Saturation effects and substitution patterns

**Figure 2:**
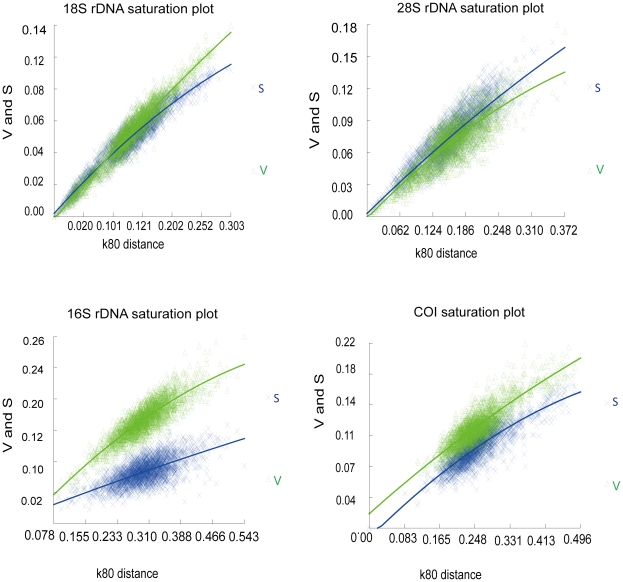
Substitution patterns of 18S rDNA, 28S rDNA, 16S rDNA and COI gene. The number of transition (S) and transversion (V) substitutions is plotted against Kimura 2 parameter (K2p) distance considering all sites. Each point represents a pairwise comparison among two taxa. (A) 18S rDNA saturation plot. (B) 28S rDNA saturation plot. (C) 16S rDNA saturation plot. (D) COI saturation plot.

Figure 2 showed the results of saturation analyses. Tests were performed for all four genes. Evaluation of transition and transversion substitution, from the scatter-plots, showed transition saturation in 16S rDNA and both transition and transversion saturation in COI.

### Phylogenetic analyses

Since coding gaps as missing data or as a fifth character are two more useful methods for incorporating insertion-deletion (indels) events into parsimony analyses and initially, we considered these two options. However, when gaps were coded as a fifth character, probably due to the overweight of the longer gaps in the alignments, the monophylies of Heteroptera and of the most infraorders each were all not supported. The parsimony results with gaps scored as a fifth character state for both the nuclear data and four-gene combined data were in File S1. So, our parsimony analyses were concentrated on treating gaps as missing data in the following text.

**Figure 3:**
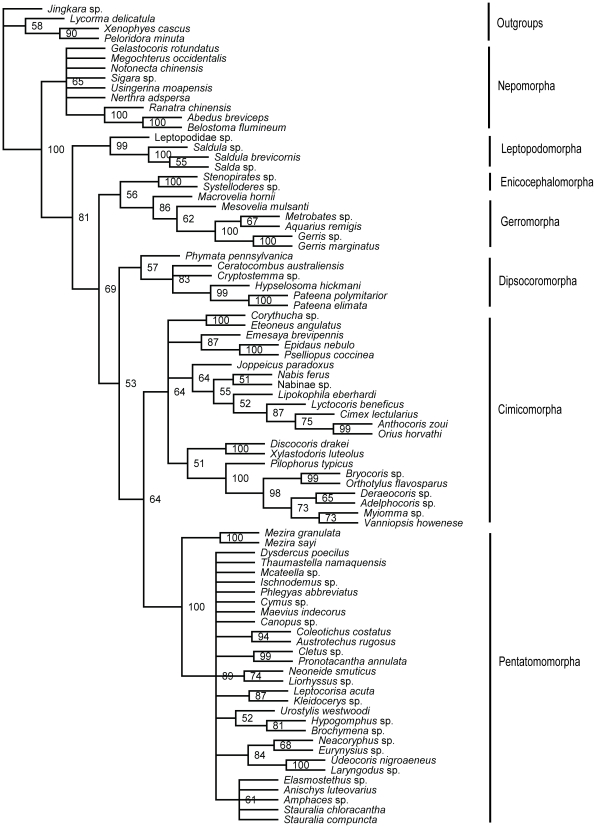
MP phylogram inferred from nuclear data set with gaps coded as missing data. Bootstrap support values (>50%) are indicated at each node.

Nuclear data set (18S+28S rDNAs)-MP tree (Figure 3): Nepomorpha was the sister group of the remaining heteropteran taxa with strong support (BP = 100%). Then Leptopodomorpha came out with moderate support value (BP = 81%). Dipsocoromorpha and (Enicocephalomorpha+Gerromorpha) were resolved as poorly supported successive sister groups to (Cimicomorpha+Pentatomomorpha) which was also weakly supported.

**Figure 4:**
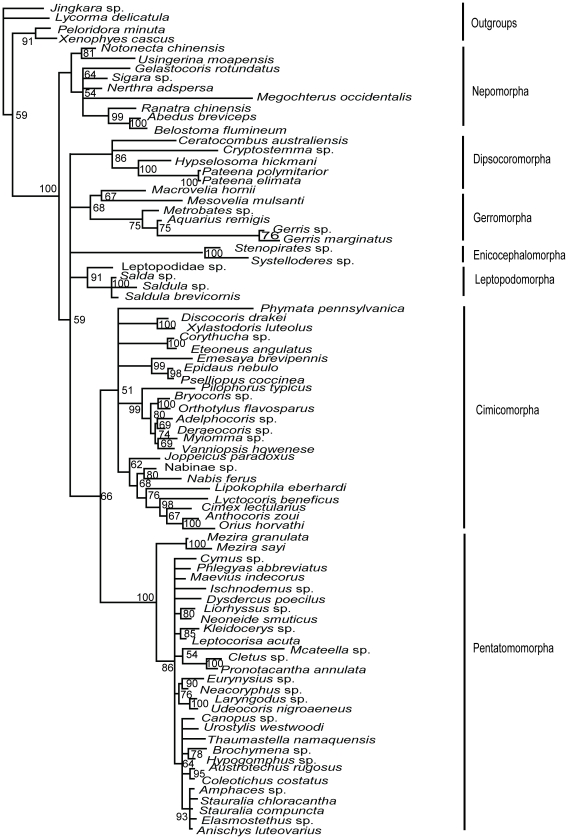
ML phylogram inferred from nuclear data set. Bootstrap support values (>50%) are indicated at each node.

Nuclear data set-ML tree (Figure 4): Nepomorpha was strongly supported as the basal heteropteran lineage (BP = 100%). Other heteropterans formed a clade in which only the sister group relationship of Cimicomorpha and Pentatomomorpha was resolved with BP value of 66%.

**Figure 5:**
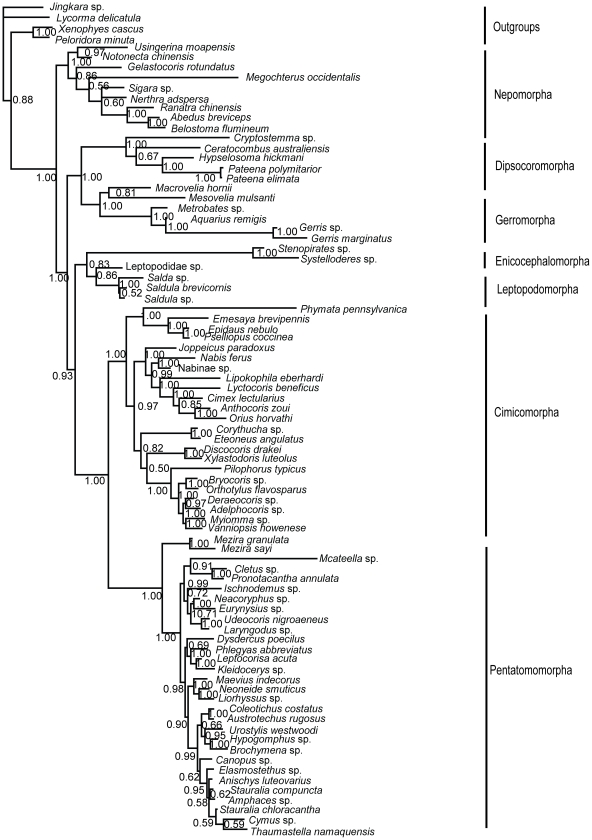
Bayesian phylogram inferred from nuclear data set. Bayesian posterior probabilities (>50%) are indicated at each node.

Nuclear data set-Bayesian tree (Figure 5): The relationships among infraorders were all well resolved in the Bayesian inference of the nuclear data. The monophyly of each of 7 infraorders was well supported. Nepomorpha was treated as the most basal clade supported by a 1.00 posterior probability. Gerromorpha and Dipsocoromorpha, Enicocephalomorpha and Leptopodomorpha, Cimicomorpha and Pentatomomorpha were formed as sister taxa with each other respectively. The relationships of these three clades were: ((Gerromorpha+Dipsocoromorpha)+((Enicocephalomorpha+Leptopodomorpha)+(Cimicomorpha+Pentatomomorpha))) and nodes were supported by 1.00, 0.83 and 1.00 PP.

Mitochondrial data set (16S rDNA+COI)-MP, ML and Bayesian tree: The monophyly of Heteroptera was not supported. The infraorders were all non-monophyletic in the MP, ML and Bayesian tree of mitochondrial data set. 16S rDNA has been proved to contain more synonymous substitution than nuclear genes [31], [32]. Besides, substitutional saturation was detected in 16S rDNA and COI in our analyses and also in Tian et al. (2008) [11] and Damgaard (2008) [8]. Obviously, 16S rDNA and COI were too fast-evolving for solving the relationships of higher taxa in our study alone (trees were not shown). Thus we preferred to base our conclusions on the nuclear trees and the combined (nuclear+mitochondrial) trees.

**Figure 6:**
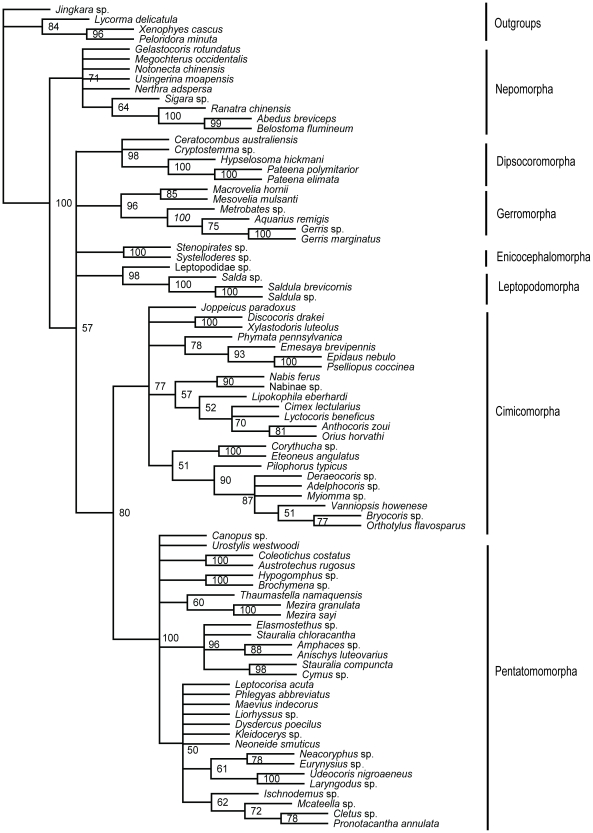
MP phylogram inferred from combined data set with gaps coded as missing data. Bootstrap support values (>50%) are indicated at each node.

Combined data set (18S+28S+16S rDNAs+COI)-MP tree (Figure 6): The basal position of Nepomorpha and the sister group relationship of Cimicomorpha and Pentatomomorpha were supported with BP values of 100% and 80%, respectively. Other four clades were resolved as polytomy.

**Figure 7:**
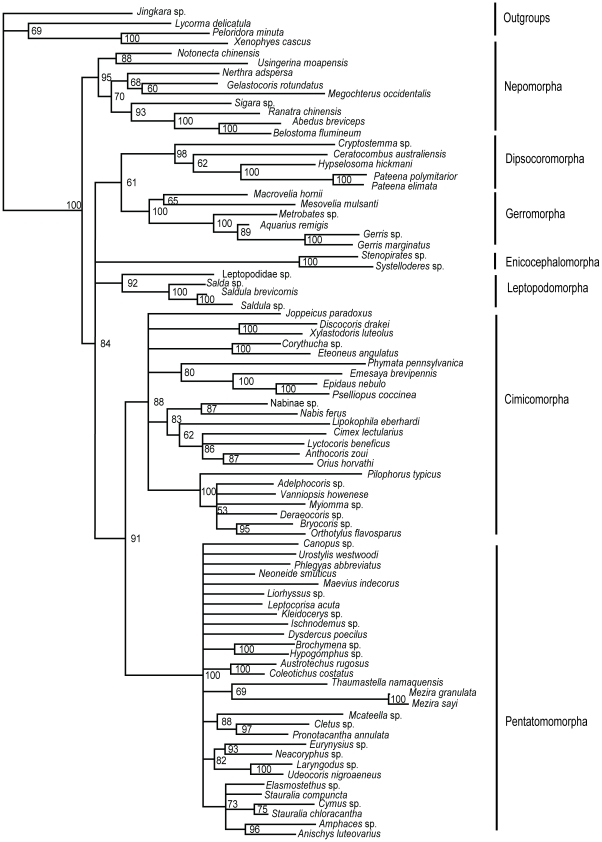
ML phylogram inferred from combined data set. Bootstrap support values (>50%) are indicated at each node.

Combined data set-ML tree (Figure 7): The maximum likelihood tree of the combined data was in accordance with the three trees (MP tree, ML tree and Bayesian tree) of nuclear data and the MP tree of the combined data that Nepomorpha was the sister group of all the remaining heteropteran taxa (BP = 100%). The other Heteroptera were divided into four lineages: (Cimicomorpha+Pentatomomorpha), (Gerromorpha+Dipsocoromorpha), Enicocephalomorpha and Leptopodomorpha. The ML tree of combined data provided a little better resolution in the relationship of (Gerromorpha+Dipsocoromorpha) than that of nuclear data although the support values were relatively low (BP = 61%). Support indices of the nodes for Cimicomorpha+Pentatomomorpha rose from 66% (ML tree of nuclear genes) to 91% (ML tree of combined data).

**Figure 8:**
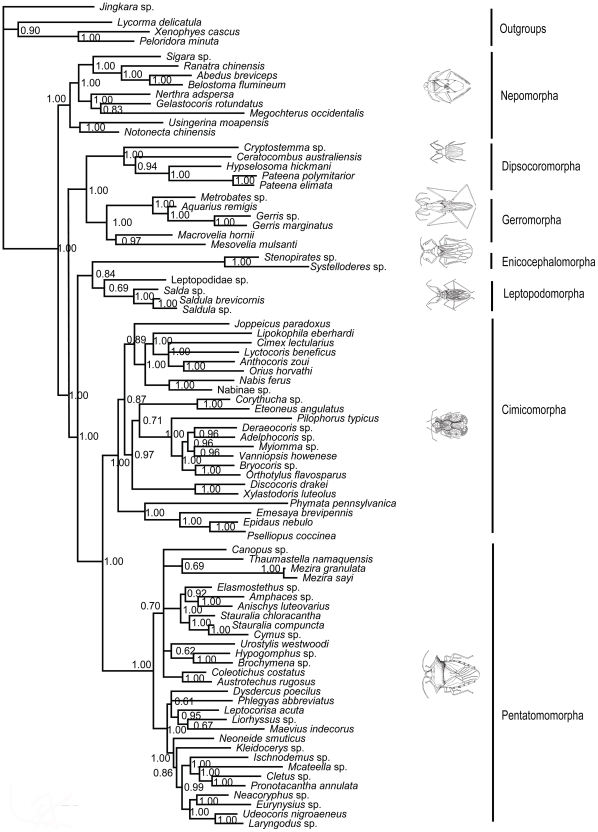
Bayesian phylogram inferred from combined data set. Bayesian posterior probabilities (>50%) are indicated at each node.

Combined data set-Bayesian tree (Figure 8): The topology of Bayesian inference of the combined data showed no difference from that of nuclear genes. Nepomorpha was the basal branch (1.00 PP), and then the sister group of (Dipsocoromorpha+Gerromorpha) (1.00 PP) came out. (Enicocephalomorpha+Leptopodomorpha) and (Cimicomorpha+Pentatomomorpha) were sister groups of each other, coming out after the sister taxa of (Dipsocoromorpha+Gerromorpha). The support value for the clade of (Enicocephalomorpha+Leptopodomorpha) was similar to that in the Bayesian tree of nuclear data.

In general, six trees generated by the three methods from two matrices were not completely alike, but with roughly similar topology. The MP, ML and Bayesian trees of combined data showed higher supports on the same nodes than those of nuclear data set. In the maximum likelihood and parsimony analyses, the ML tree of the nuclear data and the MP tree of the combined data shared the same topology; the ML tree of the combined data was similar to the above two trees with better solution in the relationship of Dipsocoromorpha and Gerromorpha; the MP tree of nuclear data was different from the above three trees in the positions of Gerromorpha, Dipsocoromorpha, Enicocephalomorpha and Leptopodomorpha. In the Bayesian analyses, the combined data and the nuclear data came to the similar results, with support values higher than in maximum parsimony and likelihood analyses. The comprehensive analyses of all results can tell that both basal clade of Nepomorpha and sister taxa relationships of Cimicomorpha and Pentatomomorpha were well supported; sister group relationships of (Gerromorpha+Dipsocoromorpha) and (Enicocephalomorpha+Leptopodomorpha), respectively, were only strongly supported in the Bayesian trees of nuclear data and combined data.

### Estimation of evolutionary time

**Figure 9:**
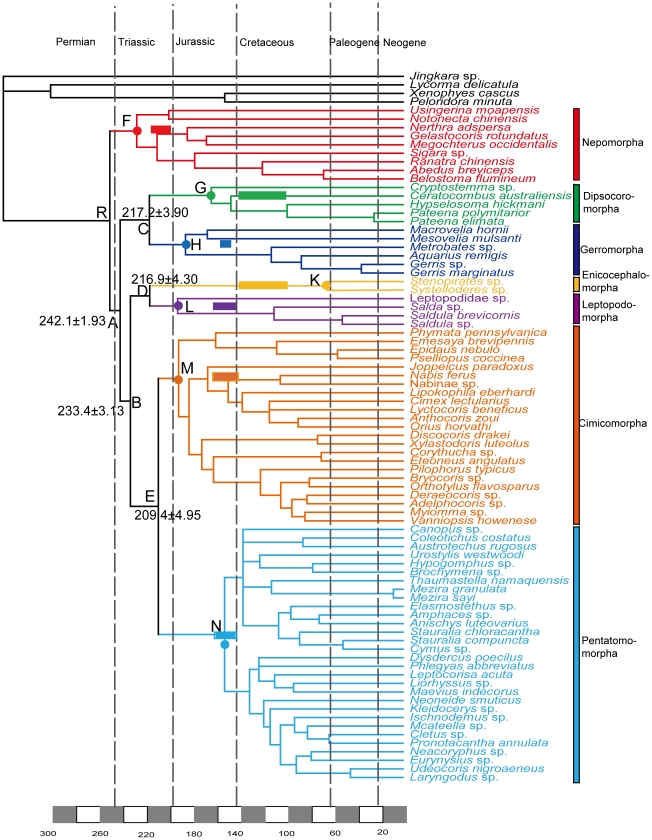
Chronogram of the maximum likelihood phylogeny of Heteroptera and outgroups generated using Penalized Likelihood. Approximate divergence dates and 95% confidence intervals of selected clades (A, B, C, D, E) are indicated along the *x*-axis. Average ages for F, G, H, K, L, M and N were: F,228.0 Mya; G,164.1 Mya; H, 186.2 Mya, K, 66.3 Mya; L,193.0 Mya; M, 192.2 Mya; N, 152.9 Mya. Bars in corresponding colours were represented the age ranges of the oldest fossils for the infraorders.

**Figure 10:**
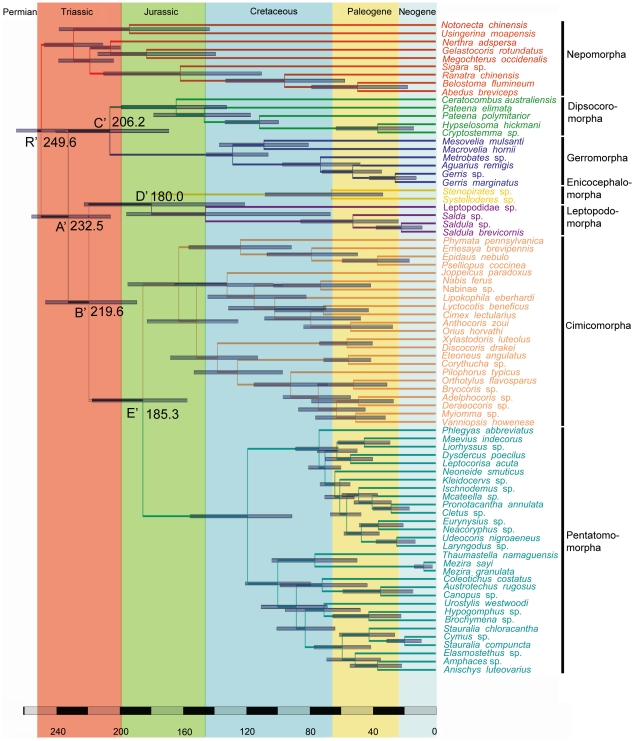
Chronogram of maximum credibility ultrametric tree the Heteroptera generated using BEAST. Branch lengths are drawn proportional to time; highest posterior density credible intervals for nodes are indicated by horizontal grey bars.

The maximum credibility tree retrieved from the combined analyses in BEAST is nearly identical to the Bayesian consensus tree in r8s. Both dating analyses suggested that all of the higher taxa within Heteroptera originated in Triassic (Figure 9 & Figure 10). In r8s analyses, Nepomorpha originated at around 251.0 Mya (node R in Figure 9) and other higher groups of Heteroptera began to diversify shortly thereafter at 242.1 Mya (node A). The divergence time of clade ((Dipsocoromorpha+Gerromorpha)+(Cimicomorpha+Pentatomomorpha)) was 233.4 Mya (node B). The optimal age estimates of infraorders Enicocephalomorpha and Leptopodomorpha implied that they originated at 217.2 Mya (node C); for that of Dipsocoromorpha and Gerromorpha was 216.9 Mya (node D). Cimicomorpha and Pentatomomorpha appeared a little later at 209.4 Mya (node E), but the major expansions of these two groups at family level were at around 62∼142 Mya in Cretaceous. For nodes R, A and B in r8s analyses, BEAST analyses gave the close date estimates as 249.6 Mya (node R′ in Figure 10), 232.5 Mya (node A′ in Figure 10) and 219.6 Mya (node B′ in Figure 10), respectively. The less deep node ages for node C′, D′ and E′ (Figure 10) were 206.2, 180.0 and 185.3 Mya, respectively. As a whole, both methods yielded broadly similar date estimates, with BEAST giving consistently shallower dates for all nodes of interest.

## Discussion

### Phylogenetic relationships of Heteroptera

Heteroptera is a monophyletic group recognized by most heteropterists [1]. Our results were in accordance with this opinion from combined data set (BP = 100; BP = 100; 1.00 PP) and nuclear data set (BP = 100; BP = 100; 1.00 PP) with high support values in MP, ML and Bayesian trees. The stable sister group relationship of Cimicomorpha and Pentatomomorpha was corroborated in our analyses. It appeared in all phylogenetic trees and especially highly supported in Bayesian inference (1.00 PP) and maximum likelihood analysis (BP = 91) of combined data sets, and the Bayesian tree of nuclear data set (1.00 PP). This clade was proposed by Schuh (1979) [16] and confirmed in research of Wheeler et al. (1993) [21], Xie et al. (2008) [23], Schuh et al, (2009) [12] and Cassis & Schuh (2010) [25].

As for the basal clade of Heteroptera, there were different views: Cobben (1968) [33] pointed out that the primitive groups of heteropteran taxa should be like Leptopodomorpha based on the characters of eggs, feeding habits and embryology. Afterwards, he proposed that the primitive clade should possess some characters of Gerromorpha [17]. Sweet (1979) [34] strongly implied that the original groups of Heteroptera should be Pentatomomorpha, but no further testification was carried out by cladistic analysis. Schuh (1979) [16] analyzed the characters from Cobben (1978) [17] with the method of cladistic analysis and arrived at a conclusion that Enicocephalomorpha was the most basal branch. This view was supported by the comprehensive work of Wheeler et al. (1993) [21] and Xie et al. (2008) [23].

In this study, Nepomorpha as the basal clade was consistently recovered in all six trees with full support of 100% BP (MP, ML trees of nuclear data and combined data), 1.00 PP (Bayesian trees of nuclear data and combine data). This was congruent with the hypothesis of Mahner (1993) [19] based on a series of morphological characters: the occurrence of three pair of tympanal organs in the two pterothoracic and the first abdominal segment; the structure of the midgut epithelium (cells with one nucleus and ileum (with U-shaped rectal gland); the spiral katatrepsis of the embryos; the presence of spiracular sieve-plates; the greatly enlarged mesepimera in Nepomorpha. Moreover, three other typical nepomorphan autapomorphies were to differentiate Nepomorpha (Cryptocerata) from the other infraorders (Gymnocerata), i.e., the short, often 3-sesmented, antennae which are folded under the head and concealed in a groove, the reduced antennal musculature and the female abdominal sternum 7 drawn out as a subgenital plate which covers the ovipositor [35]. Our result was also in accordance with that the oldest Heteroptera were nepomorphans based on fossils from the early Triassic [36], [37].

Whether Leptopodomorpha and Nepomorpha were in sister-group relationship was indefinite in former phylograms (Figure 1 A D). Here, we obtained different results: the hypotheses could not substantiate the sister group relationship of Nepomorpha and Leptopodomorpha. Leptopodomorpha was placed as sister group of Enicocephalomorpha in Bayesian trees of both nuclear data and combined data with support values of 0.83 PP, 0.84 PP, respectively. The hypopleurites of Abdominal segment eight (VIII) are prominent [22] in these two infraorders, which can be considered as an effective putative synapomorphy to connect them. The placements of Dipsocoromorpha and Gerromorpha differed from the positions inferred by Schuh (1979) [16] and Wheeler et al. (1993) [21] (Figure 1 A D). There, these two groups separated from the base of the phylogenetic trees one after another, while they were appeared as stable sister groups in our cladograms. This node was appeared in three topologies from nuclear data and combined data sets with especially high support in their Bayesian trees (1.00 PP, 1.00 PP). Two synapomorphies support this clade: the processes of the support bridge of the genitalia are undifferentiated [22]; there are very conspicuous, mostly ring-like (R structure) desclerotizations of antennal cuticle [38]. From the divergence time of these four groups (Figure 9 & Figure 10), we can see they (also Cimicomorpha and Pentatomorpha) diverged at a very short period of time (233.4∼242.1 Mya in r8s analyses; 219.6∼232.5 Mya in BEAST analyses) and that was maybe a reason for the unstable relationships of the four groups. Although there are some morphological characters to support the relationships of Enicocephalomorpha+Leptopodomorpha, Dipsocoromorpha+Gerromorpha, respectively, to obtain a more credible hypothesis of these four groups more data should be added.

### Molecular dating and evolutionary hypothesis of Heteroptera

Because both analyses gave similar estimates for the deep nodes of interest, in the subsequent discussion, we focused on estimates from the r8s analyses. From our analyses, Nepomorpha diverged earliest in early Triassic and the further diversification within Nepomorpha at superfamily or family level had lasted from middle Triassic to late Cretaceous (Figure 9). The sister groups of Enicocephalomorpha and Leptopodomorpha, Dipsocoromorpha and Gerromorpha, and Cimicomorpha and Pentatomomorpha all diverged in late Triassic and the numerous modern superfamilies in most infraorders had their origins in Jurassic. This demonstrated that the higher level radiations in the hemimetabolous heteropterans were consistent with that of the holometabolous coleopterans [28] and dipterans [29], occurred in Triassic. One explanation for this is that after the Permian-Triassic extinction events which may be caused by large or multiple bolide impact events, increased volcanism, sudden release of methane hydrates from the sea floor or increasing aridity, terrestrial ecosystems were initiated considerable transformations, e.g., many niches were left vacant [39].

According to Grimaldi & Engel, 2005 [36], the oldest nepomorphans were from the Late Triassic (199.6–228.0 Mya), including Ochteroidea, Notonectidae, Naucoridae ana Belostomatidae; aside from the possible fossil (from Late Triassic shales of Virginia) of dipsocoromorphan bugs, the oldest definitive fossils of dipsocoromorphan bugs occur in Early Cretaceous (99.6–145.5 Mya) amber from Lebanon and in mid-Cretaceous amber from Myanmar; the earliest putative leptopodomorphans was Upper Jurassic (145.5–161.2 Mya) Archegocimicidae (e.g., *Saldonia*). Otherwise, definitive fossils appear late, in Miocene amber from the Dominican Republic and Mexico; the oldest Cimicomorpha evolved in the Jurassic (145.5–199.6 Mya). For Nepomorpha, Dipsocoromorpha, Leptopodomorpha and Cimicomorpha, our molecular time estimates were around 228.0 (node F in Figure 9), 164.1(node G), 193.0 (node L), and 192.2 Mya (node M), respectively, so they fitted the fact that the molecular estimates should be older than the fossil ages. For Gerromorpha, Damgaard (2008) [40], reviewed the fossil history of semi-aquatic bugs (Gerromorpha) and inferred that the Gerromorpha probably extended back into the Triassic. Our estimated divergence age for Gerromorpha was around 186.2 Mya (node H), which was in accordance with his suggestion. For the rest two infraorders, our estimated ages were in accordance with the oldest Pentatomomorpha (∼152.9 Mya; Upper Jurassic) and earlier than the earliest known enicocephalid-like fossils which were in amber from the Early Cretaceous (99.6–145.5 Mya) of Lebanon [41], [42]. (Figure 9).

Gerromorpha and Leptopodomorpha were not showed sister-group relationship implied the semi-aquatic habitat of Gerropmopha and the damp habitat of Leptopodomorpha may occur independently; Gerromorpha separated from Dipsocoromorpha at around 217.2 Mya, implying that the water surface-dwelling originated in late Triassic.

The Cretaceous contained most of the cladogenesis at family level in our analyses, especially of Cimicomorpha and its sister group, Pentatomomorpha (Figure 9), although some fossil Cimicomorpha have been obtained from the Upper Jurassic [43], [44]. Our data suggested that most of the superfamilies representing extant cimicomorphans and pentatomorphans arose in the Jurassic as mentioned above, but began to diversify during the Cretaceous. We inferred that the rise of the angiosperm at that time was foreshadowing to the diversification of these two groups. The first flowering plants known to exist were 140 million years ago. They diversified enormously during the Lower Cretaceous and replaced conifers as the dominant trees only around 60–100 Mya [45]. The flourish of angiosperms was thought to have driven the diversification of major phytophagous groups of insects, such as beetles [28], [46] and ants [47], and it would appear that heteropterans diversification at family level closely tracked the rise of the flowering plants, too.

On the basis of our molecular dating results, we hypothesized that Nepomorpha colonized the aquatic habitat in the Triassic, the Gerromorpha and the Leptopodomorpha independently adapted to the semi-aquatic or damp habitat in the Early Jurassic. One explanation for this hypothesis was: The Permian had great diversity in insect, including the largest insects ever to have existed [48]. Scytinopteroidea, whose remains were very numerous in Late Permian and Triassic localities and were more abundant in the near-shore than off-shore, is one superfamily of Cicadomorpha. They were considered ancestral to Heteroptera [49], [50] and Nepomorpha retained more features of scytinopteroid ancestors than any other infraorder (Shcherbakov & Popov, 2002). The environmental conditions prevailing at a given time in the evolutionary history of organisms determine their evolutionary situation. The Permian-Triassic (P-T) transition has been ranked as one of most important transitions [51], i.e., the great dying (the end-Permian mass extinction). After the extinction, the ancestral Heteroptera expanded into continental waters and differentiated. During the early Triassic, the plant biomass was insufficient to form coal deposits, which implied a limited food mass for herbivores [52]. The complex ecosystems recovery began until the start of the mid-Triassic [53], then, the semiaquatic, moist habitat and terrestrial bugs started to differentiate in mid-Triassic.

In this paper, we focused primarily on the higher taxa of Heteroptera, inclusion of wider sampling and additional fossils to estimate the origin of lower taxa of extant heteropterans would be expected.

## Materials and Methods

### Taxa Sampling

As multiple species as possible were collected for each infraorder, particularly for large infraorders (e.g. Cimicomorpha, Pentatomomorpha). In total, 83 species were sampled, of which 79 species were ingroups representing 7 currently recognized infraorders [7]. Among those 83 species, 20 species were selected from the collections deposited in the Institute of Entomology, Nankai University. Sequences of the other 63 species were retrieved from GenBank. Most infraorders have more than three representatives except Enicocephalomorpha (*Stenopirates* sp., *Systelloderes* sp.). One species of Membracidae (Hemiptrea: Cicadomorpha), one species of Fulgoridae (Hemiptera: Fulgoromorpha) and two species of Peloridiidae (Hemiptera: Coleorrhyncha) were selected as outgroups in this work. The details were shown in File S2.

### DNA extraction, PCR amplification and DNA sequencing

DNA was extracted from a single individual of each species preserved in 95% ethanol at −20°C, which followed the modified CTAB procedure [54]. Heads, abdomens and wings were removed and kept in 95% ethanol as voucher specimens. The complete 18S rDNA and the D3 region of 28S rDNA, and parts of two mitochondrial genes, 16S rDNA and COI, were amplified and sequenced using primers listed in File S3.

PCR reactions took place in 25 µl volumes containing 14.2 µl of ddH_2_O, 1 µl of dNTP (2.5 mM) (TAKARA, Dalian, China), 2.5 µl of buffer (10×), 3.0 µl of MgCl_2_ (25 mM), 1 µl of primer each (10 µM), and 0.3 µl of Taq (2.5 Units) (TIANGEN, Beijing, China). PCR conditions were: 94°C for 1 min, followed by 35 cycles of 94°C 30 s, 45–50°C 30 s, 72°C 1 min, and finally 72°C for 10 min. Double-strand PCR products were amplified in a TGRADIENT gradient (Biometra, German). Target products were purified with Agarose Gel DNA Purification Kit (TAKARA, Dalian) and sequenced on both strands with the same primers used for PCR reaction by SunBio Company.

### Sequence alignment

Edited sequence contigs for all four genes were aligned using the software program Clustal X [55], BioEditor 1.6.1 [56] and MEGA 4.0 [57] with default settings. To avoid bias in refining alignments, ambiguous alignment positions including beginning and end regions were excluded by BioEditor 1.6.1 [56]. Further, COI nucleotide sequences were translated into amino acid sequences using invertebrate mitochondrial code in MEGA 4.0 to prove the correctness of DNA sequences. Pairwised distance and nucleotide substitutions were also calculated by implementing MEGA 4.0.

### Saturation test

Because saturation in substitutions could lead to incorrect phylogenetic inferences [58], each gene was evaluated for transition and transversion substitutions by DAMBE V4.5.32 [59].

### Phylogenetic analyses

Phylogenetic analyses using maximum parsimony was performed by PAUP 4.0 b windows version [60]. Characters were equally weighted and alignment gaps (indels) were treated as missing data and as a fifth character. For MP analyses, heuristic searches were performed with 10 random-taxa-addition replicates and the strategy TBR (Tree Bisection and Reconnection) branch swapping. Bootstrap values (BP) [61] were calculated with 1000 replicates. Analyses were performed for the nuclear data set and the mitochondrial data, as well as the all-combined data set. Trees were displayed with TREEVIEW 1.6.6 [62].

The maximum likelihood methods were conducted with TreeFinder [63]. The substitution models of 18S rDNA, 28S rDNA, 16S rDNA and COI were all GTR [Optimum, Empirical]: G [Optimum]:5, proposed by TreeFinder. In ML analysis, the parameters were estimated during analyses and the support for the likelihood-derived topologies was estimated by bootstrap resampling (BP) [61] calculated using 1000 replicates.

Bayesian inference of likelihood [64], [65] was implemented in MrBayes 3.1.1 [66] for the nuclear data and all the combined data sets. Best-fitting nucleotide substitution models for each gene were selected by Modeltest 3.7 [67] using the Akaike Information Criterion (AIC). Two-parallel Markov chain Monte Carlo sampling was performed with four chains each. This analysis was continued until the standard deviation of split frequencies was below 0.01. The most appropriate substitution model was GTR+I+G for 18S rDNA and 16 rDNA, SYM+I+G for 28S rDNA and TVM+I+G for COI. It ran 100 million generations for the nuclear data, and 150 million generations for the combined data set. Convergence and stabilization of all parameters were visually inspected and verified using the program Tracer V1.5 [68]. Topologies prior to the negative natural logarithm of likelihood equilibrium were discarded as “burn-in” and clade posterior probabilities (PP) were computed from the remaining trees. (Date matrixes can be obtained in File S4).

### Molecular dating

Divergence date estimates were calculated for higher level groups of Heteroptera using two methods: a penalized likelihood (PL) method using the software r8s, V1.7.1 [69], and a Bayesian MCMC approach using the software BEAST V1.5.3 [68].

#### r8s analysis

The tree obtained by Bayesian inference including branch length was used as a fixed input tree for divergence time estimation in the programme r8s V1.7.1 [70]. Cross-validation method in r8s was used to determine the optimal level of rate-smoothing of the PL analyses with smoothing parameters varying from 1 to 1×10^3^. We used a smoothing parameter of 100 for these data. To estimate the 95% confidence intervals of the divergence dates, we constructed 100 bootstrap data sets of the combined data using SEQBOOT from the PHYLIP V3.6 [71]. Branch lengths were estimated from each bootstrapped dataset using the DNAML module also in PHYLIP V3.6. We used the following fossils as calibration points: The most recent common ancestor of the clade including *Hypselosoma hickmani* Wygodzinsky, 1959, *Pateena polymitarior* Hill, 1980 and *Pateena elimata* Hill, 1980 was constrained to be at least 99.6 Mya (minage = 99.6) based on amber *Libanohypselosoma popovi* Azar & Nel, 2010, the earliest record of the Schizopteridae [72]; The most recent common ancestor of the clade Gerridae including *Metrobates sp.*, *Aquarius remigis* Say, *Gerris marginatus* Say, 1832 and *Gerris* sp. was constrained to be at least 55.0 Mya (minage = 55.0) based on amber *Cretogerris albianus* Perrichot, Nel & Neraudeau, 2005 [40], [73]; The most recent common ancestor of the clade Ochteroidea including *Megochterus occidentalis* Baehr, 1990, *Gelastocoris oculatus* (Fabricius, 1798) and *Nerthra adspersa* (Stål, 1863) was constrained to be at least 199.6 Mya (minage = 199.6) according to Grimaldi & Engel, 2005 [36]; The most recent common ancestor of the clade including *Discocoris drakei* Slater & Ashlock, 1959 and *Xylastodoris luteolus* Barber, 1920 was constrained to be at least 55.8 Mya (minage = 55.8) based on amber *Protodoris minusculus* Nel, 2004, the oldest fossil of Thaumastocoridae [74]. The root was fixed at 251 Mya, according to the approximate age of the earliest fossil of Heteroptera: *Paraknightia magnifica* Ev., 1943 thought to be of latest Permian [36].

#### BEAST analysis

BEAST uses the aligned sequence data to generate a tree in the MCMC (Markov chain Monte Carlo) process to infer divergence times. The unlinked GTR model of nucleotide substitution, gamma-distributed rate variation and a proportion of invariant sites of heterogeneity model were applied with base frequencies estimated during the analysis. A relaxed molecular clock using the uncorrelated log-normal model was applied with a Yule process speciation prior for branching rates. The final analysis consisted of two independent MCMC analyses; each chain was run for 10 million generations (burn-in 10%) with parameters sampled every 1000 steps to ensure effective sample size (ESS) values above 200 for most parameters. Independent runs were combined using LogCombiner V1.5.3 [57]. Tracer V1.5 [57] was used to confirm adequate mixing of the MCMC chain, and FigTree V1.3.1 [75] was used to visualize the consensus tree along with node ages and age deviations. Outgroup taxa were not presented for the molecular dating analysis. To maximize similarity with the r8s analyses, we used the same calibration points in BEAST analyses. We used calibration nodes with uniform priors bounded between 99.6–145.5 and 199.6–228.0 Mya for Schizopteridae and Ochteroidea, respectively. The most recent common ancestor of the clade of Gerridae and Thaumastocoridae was given a normally distributed prior with a mean age of 55.0, 55.8 and a standard deviation of 8.0, 10.0, respectively. The root node was limited to a mean age of 251 Mya with a standard deviation of 7 Mya based on the estimated age of the suborder [36] (Detailed information in the above *r8s analysis*).

## Supporting Information

File S1 **MP phylograms for nuclear data set and combined data set with gaps coded as a fifth character.** Figure S1, MP phylogram inferred from nuclear data set. Bootstrap support values (>50%) are indicated at each node. Figure S2, MP phylogram inferred from combined data set. Bootstrap support values (>50%) are indicated at each node.
(RAR)

File S2 **Taxa used in this study.**
(DOC)

File S3 **Genes included in study, primer sequences and sources and locus-specific annealing temperatures (TA).**
(DOC)

File S4 **Date matrixes of this study.**
(RAR)
