# Supplementary material for: Higher Level Phylogeny and the First Divergence Time Estimation of Heteroptera (Insecta: Hemiptera) Based on Multiple Genes
Source: PLoS One. 2012 Feb 27;7(2):e32152. doi: 10.1371/journal.pone.0032152 (PMC3288068; doi:10.1371/journal.pone.0032152)
Supplement: File S2 — Taxa used in this study. (DOC) [file pone.0032152.s002.doc]

### Taxa used in this study.

| **current family classification** | | **Taxon** | **Accession number** | | | |
| --- | --- | --- | --- | --- | --- | --- |
| **Infraorder** | **Family** | **Species** | **COI** | **18S rDNA** | **28S rDNA** | **16S rDNA** |
| Cimicomorpha | Miridae | *Myiomma* sp. | AY253124 | AY252404 | AY252626 | AY252885 |
|  |  | *Deraeocoris* sp. | AY252235 | AY252235 | AY252478 | AY252848 |
|  |  | *Vanniopsis howenese* | FJ226450 | FJ226442 | FJ226447 | FJ226439 |
|  |  | *Bryocoris* sp. | JQ004041 | JQ004029 | JQ004033 | JQ004037 |
|  |  | *Orthotylus flavosparus* (Sahlberg, 1841) | JQ004042 | JQ004030 | JQ004034 | JQ004038 |
|  |  | *Adelphocoris* sp. | JQ004043 | JQ004031 | JQ004035 | JQ004039 |
|  |  | *Pilophorus typicus* (Distant, 1909) | JQ004044 | JQ004032 | JQ004036 | JQ004040 |
|  | Anthocoridae | *Anthocoris zoui* (Bu & Zheng, 2001) | HQ407467 | EF487303 | EF487330 | EF487275 |
|  |  | *Orius horvathi* (Reuter, 1884) | HQ407468 | EF487299 | EF487327 | EF487278 |
|  | Nabidae | *Nabis ferus (*Linnaeus, 1758) | HQ407469 | EF487300 | EF487332 | EF487281 |
|  |  | Nabinae sp. | EU683241 | EU683162 | EU683206 | AY252703 |
|  | Reduviidae | *Emesaya brevipennis* (Say, 1832) | AY253047 | AY252321 | AY252560 | AY185832 |
|  |  | *Epidaus nebulo* (Stal, 1863) | HQ407470 | EF487305 | EF487328 | EF487279 |
|  |  | *Pselliopus coccinea* | AY252970 | AY252230 | AY252473 | AY252701 |
|  |  | *Phymata pennsylvanica* Handlirsch, 1897 | EF641150 | AY252285 | AY252531 | AY252758 |
|  | Tingidae | *Corythucha* sp. | AY253013 | AY252284 | AY252530 | AY252757 |
|  |  | *Eteoneus angulatus* Drake et Maa | EF523481 | EF487311 | EF487321 | EF487290 |
|  | Cimicidae | *Cimex lectularius* Linnaeus, 1758 | AY253011 | AY252281 | AY252526 | AY252754 |
|  | Joppeicidae | *Joppeicus paradoxus* Putton, 1881 | AY252951 | AY252206 | AY252455 | AY252688 |
|  | Thaumastocoridae | *Discocoris drakei* Slater & Ashlock, 1959 | EU683229 | EU683137 | EU683196 | EU683092 |
|  |  | *Xylastodoris luteolus* Barber, 1920 | EU683252 | FJ226443 | EU683216 | EU683109 |
|  | Lyctocoridae | *Lyctocoris benefices* (Hiura 1957) | GQ292284 | EF487298 | EF487324 | EF487282 |
|  | Plokiophilidae | *Lipokophila eberhardi* Schuh 1993 | EU683235 | EU683149 | EU683202 | EU683096 |
| Dipsocoromorpha | Dipsocoridae | *Ceratocombus australiensis* Gross, 1950 | AY253029 | AY252300 | AY252547 | AY252775 |
|  |  | *Cryptostemma* sp. | AY253030 | AY252301 | AY252548 | AY252776 |
|  | Schizopteridae | *Hypselosoma hickmani* Wygodzinsky, 1959 | AY253033 | AY252304 | AY252550 | AY252779 |
|  |  | *Pateena polymitarior* Hill, 1980 | AY253031 | AY252302 | AY252549 | AY252777 |
|  |  | *Pateena elimata* Hill, 1980 | AY253032 | AY252303 | No | AY252778 |
| Enicocephalomorpha | Enicocephalidae | *Stenopirates* sp. | HQ407475 | FJ372645 | FJ372664 | FJ372638 |
|  |  | *Systelloderes* sp. | EU871270 | AY252415 | EU871209 | EU871149 |
| Gerromorpha | Gerridae | *Metrobates* sp. | AY252971 | AY252232 | AY252475 | AY252704 |
|  |  | *Gerris* sp. | HQ407471 | FJ372641 | FJ372660 | FJ372633 |
|  |  | *Gerris marginatus* Say, 1832 | AY252904 | AY252122 | DQ683338 | AY252646 |
|  |  | *Aquarius remigis* Say | AF200251 | ARU15691 | AY648158 | AY425190 |
|  | Macroveliidae | *Macrovelia hornii* Uhler, 1872 | AY252946 | AY252196 | AY252450 | AY252683 |
|  | Mesoveliidae | *Mesovelia mulsanti* White, 1879 | AY252905 | AY252123 | EU871213 | EU871153 |
| Leptopodomorpha | Saldidae | *Saldula brevicornis* Rimes, 1951 | No | AY252416 | AY252635 | AY252894 |
|  |  | *Saldula* sp. | HQ407465 | FJ372639 | FJ372658 | FJ372631 |
|  |  | *Salda* sp. | EU683247 | EU683178 | EU683209 | EU683107 |
|  | Leptopodidae | Leptopodidae sp. | HQ407466 | FJ372640 | FJ372659 | FJ372632 |
| Nepomorpha | Belostomatidae | *Abedus breviceps* Stål, 1862 | AY252941 | AY252186 | AY252440 | AY252676 |
|  |  | *Belostoma flumineum* Say, 1832 | AY252913 | AY252132 | No | AY252651 |
|  | Nepidae | *Ranatra chinensis* (Mayr, 1865) | HQ407474 | FJ372644 | FJ372663 | FJ372637 |
|  | Ochteridae | *Megochterus occidentalis* Baehr, 1990 | AY253010 | EF641197 | AY252525 | AY252753 |
|  | Gelastocoridae | *Gelastocoris oculatus* (Fabricius, 1798) | AY252949 | AY252140 | AY252453 | AY585760 |
|  |  | *Nerthra adspersa*  [(Stål, 1863)](http://www.discoverlife.org/mp/20q?search=Nerthra+adspersa) | AY253009 | AY252280 | AY252524 | AY252752 |
|  | Corixidae | *Sigara* sp. | HQ407472 | FJ372642 | FJ372661 | FJ372634 |
|  | Naucoridae | *Usingerina moapensis* La Rivers, 1950 | AY252942 | AY252189 | AY252443 | AY252677 |
|  | Notonectidae | *Notonecta chinensis* Fallou, 1887 | HQ407473 | FJ372643 | FJ372662 | FJ372635 |
| Pentatomomorpha | Aradidae | *Mezira granulata* (Say, 1832) | AY252962 | AY252221 | No | AY252694 |
|  |  | *Mezira sayi* Kormilev, 1982 | EU683238 | EU683157 | EF641177 | EU683100 |
|  | Alydidae | *Leptocorisa acuta* (Thunberg) | AY252956 | AY627322 | AY252462 | AY252691 |
|  | Coreidae | *Cletus* sp. | AY252993 | AY252261 | AY252504 | AY252734 |
|  |  | *Liorhyssus* sp. | AY252945 | AY252194 | AY252448 | AY252682 |
|  |  | *Maevius indecorus* Stål, 1874 | AY252957 | AY252214 | AY252463 | AY252692 |
|  | Berytidae | *Neoneides muticus* (Say 1832) | AY253130 | AY252412 | AY252631 | AY252892 |
|  |  | *Pronotacantha annulata* (Uhler, 1893) | AY253114 | AY252393 | AY252615 | AY252874 |
|  | Cymidae | *Cymus* sp. | AY252996 | AY252264 | AY252445 | AY252738 |
|  | Lygaeidae | *Kleidocerys* sp. | AY253128 | AY252410 | AY252629 | AY252890 |
|  |  | *Neacoryphus* sp. | AY253129 | AY252411 | AY252630 | AY252891 |
|  |  | *Eurynysius* sp. | AY252991 | AY252193 | AY252502 | AY252732 |
|  | Rhyparochromidae | *Udeocoris nigroaeneus* (Erichson) | AY252994 | AY252262 | AY252505 | AY252735 |
|  |  | *Laryngodus* sp. | AY252943 | AY252192 | AY252446 | AY252680 |
|  |  | *Phlegyas abbreviates* (Uhler, 1876) | AY253017 | AY252289 | AY252535 | AY252762 |
|  | Blissidae | *Ischnodemus* sp. | AY252950 | AY252204 | AY252454 | AY252687 |
|  | Piesmatidae | *Mcateella* sp. | AY252992 | AY252260 | AY252503 | AY252667 |
|  | Pyrrhocoridae | *Dysdercus poecilus* | AY627337 | AY627318 | AY252563 | AY252799 |
|  | Acanthosomatide | *Elasmostethus* sp. | AY253048 | AY252322 | AY252561 | AY252797 |
|  |  | *Stauralia compuncta* Bergroth, 1895 | AY253001 | AY252269 | AY252513 | AY252741 |
|  |  | *Stauralia chloracantha* Dallas, 1851 | AY253000 | AY252268 | AY252512 | AY252740 |
|  |  | *Amphaces* sp. | AY253002 | AY252271 | AY252515 | AY252743 |
|  |  | *Anischys luteovarius* (Westwood, 1837) | AY253003 | AY252272 | AY252516 | AY252744 |
|  | Canopidae | *Canopus* sp. | AY252969 | AY252229 | AY252472 | AY252700 |
|  | Pentatomidae | *Hypogomphus* sp. | AY253007 | AY252278 | AY252522 | AY252750 |
|  |  | *Austrotechus rugosus* | AY253004 | AY252273 | AY252517 | AY252745 |
|  |  | *Brochymena* sp. | AY252918 | AY252137 | EF641187 | AY252655 |
|  | Scutelleridae | *Coleotichus costatus* (Fabricius, 1787) | AY253005 | AY252274 | AY252518 | AY252746 |
|  | Thaumastellidae | *Thaumastella namaquensis* Schaeffer & Wilcox 1971 | EF641176 | EF641222 | EF641196 | |EF641148 |
|  | Urostylididae | *Urostylis westwoodi* Scott | AY252952 | AY252207 | AY252456 | AY252689 |
| *Outgroup* |  | | | | | |
| Cicadomorpha | Membracidae | *Jingkara* sp. | HQ407476 | FJ372647 | FJ372666 | FJ372636 |
| Fulgoromorpha | Fulgoridae | *Lycorma delicatula* (White, 1845) | HQ407477 | FJ372646 | FJ372665 | AF158058 |
| Coleorrhyncha | Peloridiidae | *Xenophyes cascus* Bergroth, 1924 | JN546580 | JN626902 | JN626904 | JN546579 |
|  |  | *Peloridora minuta* China, 1962 | HM017506 | HM017319 | HM017427 | No |

Note: Taxa, whose sequence accession number started with FJ, EF, HQ and JQ are sequence got from this research. Other sequences are from GenBank (e.g. Schuh et al., 2009). ‘‘No”, No sequence.

*Dysdercus poecilus* 18S rDNA (AY627318) has been combined with *Dysdercus* sp. COI (AY252966), 28S rDNA (AY252563) and 16S rDNA (AY252799) in analyses.

*Systelloderes* sp. 18S rDNA (AY252415) has been combined with *Systelloderes* sp. COI (EU871270), 28S rDNA (EU871209) and 16S rDNA (EU871149) in analyses.
