# Supplementary material for: Higher Level Phylogeny and the First Divergence Time Estimation of Heteroptera (Insecta: Hemiptera) Based on Multiple Genes
Source: PLoS One. 2012 Feb 27;7(2):e32152. doi: 10.1371/journal.pone.0032152 (PMC3288068; doi:10.1371/journal.pone.0032152)
Supplement: File S3 — Genes included in study, primer sequences and sources and locus-specific annealing temperatures (TA). (DOC) [file pone.0032152.s003.doc]

Genes included in study, primer sequences and sources and locus-specific annealing temperatures (TA).

| **Gene** | **Primer name and sequence(5’-3’)** | **Primer source** | **TA** |
| --- | --- | --- | --- |
| 18S rDNA | 18S P1:CTGGTTGATCCTGCCAGTAGT | Campell et al., 1995 | 48-50°C |
|  | 18S P3:GGTTAGAACTAGGGCGGTATCT | Campell et al., 1995 |  |
| 18S rDNA | 18S P2:AGATACCGCCCTAGTTCTAACC | Campell et al., 1995 | 45-50°C |
|  | 18S P4:GATCCTTCTGCAGGTTCACC | Campell et al., 1995 |  |
| 28S rDNA | 28S DD: GGGACCCGTCTTGAAACAC | Hillis and Dixon，1991 | 48°C |
|  | 28S FF: TTACACACTCCTTAGCGGAT | Hillis and Dixon，1991 |  |
| COI | COI 1:GGAACAGGATGAACAGTTTACCCTCC | Simon et al., 1994 | 50°C |
|  | COI 2:TCTGAATATCGTCGAGGTATTC | Simon et al., 1994 |  |
| 16S rDNA | 16S A: CGCCTGTTTAACAAAAACAT | Simon et al., 1994 | 50-55°C |
|  | 16S B: CCGGTTGAACTCAGATCA | Kambhampati and Smith, 1994 |  |

Note: The complete 18S rDNA was amplified and sequenced in two contiguous regions using primer pairs 18S P1/18S P3 and 18S P2/18S P4.
